# Gamma knife radiosurgery for elderly patients with brain metastases: evaluation of scoring systems that predict survival

**DOI:** 10.1186/s12885-015-1070-y

**Published:** 2015-02-14

**Authors:** Jae-Young Park, Kyung-Sub Moon, Kyung-Hwa Lee, Sa-Hoe Lim, Woo-Youl Jang, Hyeseon Lee, Tae-Young Jung, In-Young Kim, Shin Jung

**Affiliations:** 1 Department of Neurosurgery, Chonnam National University Research Institute of Medical Sciences, Chonnam National University Hwasun Hospital & Medical School, Gwangju, South Korea; 2 Department of Pathology, Chonnam National University Research Institute of Medical Sciences, Chonnam National University Hwasun Hospital & Medical School, Gwangju, South Korea; 3 Department of Industrial & Management Engineering, Pohang University of Science and Technology, Pohang, South Korea

**Keywords:** Brain metastasis, Elderly, Gamma knife radiosurgery, Prognosis, Survival

## Abstract

**Background:**

Gamma knife radiosurgery (GKRS) has been increasingly employed for the treatment of elderly patients with brain metastases, mainly due to its demonstrated effectiveness and low complication rate. However, only a few studies have investigated the prognostic factors that influence the survival of elderly patients after GKRS. The purpose of this study was to identify a scoring system that is able to predict the survival of elderly patients undergoing GKRS using data obtained at the time of diagnosis for brain metastases.

**Methods:**

Between 2004 and 2011, death was confirmed in 147 patients aged 70 years and older who had been treated with GKRS for brain metastases. Median age at the time of GKRS was 75.7 years (range, 70–86 years). The median tumor volume was 5.1 cm^3^ (range, 0.05–59.9 cm^3^). The median marginal prescription dose was 21.4 Gy (range, 14–25 Gy).

**Results:**

The median survival was 167 days. Overall survival rates at 6 months and 1 year were 60.4% and 29.4%, respectively. Among the patient characteristics pertaining to systemic cancer and brain metastasis for which data were obtained preoperatively, a multivariate analysis showed that low Karnofsky performance status (KPS ≤ 80, *P* = 0.047) and the presence of extracranial metastases (*P* = 0.014) detected at the time of brain metastasis diagnosis were independent prognostic factors for short survival. A high score index for radiosurgery (SIR score ≥ 4, *P* = 0.024) and a high graded prognostic assessment (GPA score ≥ 2, *P* = 0.004) were associated with longer survival. A multivariate analysis of the important characteristics of systemic cancer, and the scoring system evaluating survival duration showed that a low GPA score was the most powerful independent factor for predicting short survival (hazard ratio 1.756, 95% confidence interval 1.252–2.456, *P* = 0.001).

**Conclusions:**

GKRS is a safe approach to treat brain metastases in patients age 70 years and older. In this group, our study identified GPA score at the time of GKRS as a powerful prognostic factor for survival.

## Background

Metastatic brain cancer is almost ten times more common than a primary malignant brain tumor and 20-40% of cancer patients will be diagnosed with a metastatic brain tumor [1]. If these patients are left untreated, the median survival time is 1–2 months [2], with a 1-year survival rate of 10.4% [3].

The incidence of cancer increases with age. In 2000, 12.6–18.1% of the population in developed countries was over 65 years of age [4]. In South Korea, the crude incidence rate of cancer development in this age group is 1,606 cases per 100,000 individuals [5]. However, with advances in imaging and chemotherapy, the detection and treatment of cancer, and thus the life expectancy of elderly cancer patients has improved. Among those with brain metastases, conventional treatment methods currently include surgical resection, whole-brain radiotherapy (WBRT), stereotactic radiosurgery (SRS), or a combination thereof [6]. However, selection of the most suitable therapy is difficult and must consider factors such as patient’s age, neurologic performance, systemic disease status, and the size, volume, location, and number of metastases at presentation [1]. Advanced age is a poor prognostic factor for survival in patients with brain metastases [7], and the choice of treatment is complicated by the fact that elderly patients often have multiple, concurrent diseases that can restrict their physiological reserve and physical functioning.

Although WBRT has been generally accepted as a standard treatment for several decades, accumulated evidence suggests its association with a higher risk of neurocognitive deterioration in elderly patients with brain metastases [8,9]. Thus, as an alternative approach, gamma knife radiosurgery (GKRS) has gained increasing favor as the primary treatment modality [10]. The purpose of this study was to identify a scoring system able to predict survival outcome in patients age 70 years and older who underwent GKRS for brain metastases. The predictive power of four different scoring systems was evaluated: graded prognostic assessment (GPA), recursive portioning analysis (RPA), the score index for radiosurgery (SIR), and the basic score for brain metastases (BSBM) [11-15].

## Methods

### Patients

The study was conducted in compliance with the Declaration of Helsinki (sixth revision, 2008), and fulfilled all of the requirements for patient anonymity. This study was approved by the Institutional Review Board of the Chonnam National University Hwasun Hospital (CNUHH-2014-31). A database of patients with brain tumors treated at our institution was used to identify the 1174 patients with brain metastasis who underwent GKRS between May 2004 and December 2013. From this group, the 320 patients older than 70 years of age were selected and their data were reviewed. Patients previously treated with WBRT were excluded from this study. Among the included patients, there were 147 confirmed deaths. These patients were the focus of this study.

### Analysis variables

The clinical and radiological data of the patients at the time of diagnosis of brain metastasis were collected. Clinical data included age, sex, presenting symptoms, time interval between the diagnosis of primary cancer and brain metastasis, Karnofsky performance status (KPS), and survival time. Radiological data included the presence of extracranial metastasis, the status of the primary cancer, the number and location of brain lesions, the size or volume of the largest brain lesion, and concomitant intratumoral hemorrhagic changes. Based on both sets of data, RPA, GPA, SIR, and BSBM scores were calculated. The RPA classification assigns patients with brain metastases to one of three classes that predict survival [15]: Class I patients are those with a KPS ≥ 70 at an age < 65 years with controlled primary disease and no evidence of extracranial metastases. Class III patients have a KPS < 70. Class II patients are those who do not fit into classes I or III. The GPA classification considers age, KPS, the presence of extracranial metastases, and the number of brain metastases [13]. The SIR uses a system of seven grades to determine prognosis based on age, KPS, primary cancer status, number of brain metastases, and volume of the largest brain metastasis [12]. The parameters of the BSBM classification are the KPS, primary cancer status, and the presence of extracranial metastases [14]. The features of the scoring systems used in this study are summarized in Table 1.

**Table 1:** **Prognostic scoring systems (GPA, SIR, BSBM)**

	GPA score			SIR score			BSBM score	
	0	0.5	1	0	1	2	0	1
Age	≥60	51-59	≤50	≥60	51-59	≤50	NA	
KPS	<70	70-80	90-100	≤50	60-70	>70	50-70	80-100
Control of primary cancer	NA			PD	PR-SD	CR-NED	No	Yes
Volume of the largest BM (cc)	NA			>13	5-13	<5	NA	
No. of BM	>3	2-3	1	≥3	2	1	NA	
EC metastasis	(+)		(−)	NA			(+)	(−)

BM; brain metastasis, CR; complete regression, EC; extracranial, KPS; Karnofsky performance status, NA; not available, NED; no evidence of disease, No.; number, PD; progression disease, PR; partial regression, SD; stable disease.

### GKRS protocol for brain metastasis

GKRS, performed using the Leksell Gamma Knife (model C or Perfexion, Elekta AB, Stockholm, Sweden), was used to treat 455 lesions in the 147 patients included in this study. The median maximal dose was 37 Gy (range, 18–62.5 Gy), with a median marginal tumor dose of 21 Gy (range, 14–25 Gy) at the 40–85% isodose line.

### Statistical analysis

Overall survival (OS) was defined as the time between the dates of brain metastasis diagnosis until death. The probability of OS was analyzed according to the Kaplan-Meier method, and the resulting values were compared using log-rank tests. Factors considered to be predictive of OS were analyzed using a multivariate logistic regression model. All of the statistical analyses were performed using SPSS version 20.0 for Windows (SPSS, Chicago, IL, USA); *P* < 0.05 considered statistically significant.

## Results

### Patient characteristics

The clinicoradiological characteristics of the enrolled patients are summarized in Table 2. The most common presenting symptoms were motor/sensory deficits, headache, and dizziness. Major neurological symptoms, such as sensory/motor deficit, deterioration of mental status, gait disturbance, or swallowing difficulty, were detected in 53 patients (36.1%). The primary cancer site was the lung (n = 111, non-small-cell lung cancer in 93 patients and small-cell lung cancer in 18 patients). The median time between the diagnosis of primary cancer and that of brain metastasis was 11.4 months (range, 0–106 months). Brain metastases were synchronously (within 3 months after the diagnosis of the primary cancer) detected in 65 patients (44.2%).

**Table 2:** **Summary of tumor characteristics and treatment parameters**

Parameter	No. (%)
Characteristics of systemic course	
Number of patients	147
Sex	
Female	39 (26.6%)
Male	108 (73.4%)
Age	
Median (range)	75.6 (70–86)
70 - 75	80 (54.4%)
>75	67 (45.6%)
Signs and symptoms	
Mental status change	10 (6.8%)
Dizziness	12 (8.2%)
Motor/sensory deficit	40 (27.3%)
Gait disturbance	1 (0.7%)
Headache	27 (18.3%)
Nausea & vomiting	2 (1.3%)
Swallowing difficulty	2 (1.3%)
Incidental detect	53 (36.1%)
Karnofsky Performance Status (KPS)	
Median (range)	84 (40–100)
≤60	21 (14.2%)
70-80	41 (28.0%)
90≤	85 (57.8%)
Primary tumor	
Lung cancer	111 (75.5%)
Rectal cancer	7 (4.7%)
Colon cancer	6 (4.0%)
Gastric cancer	3 (2.0%)
Breast cancer	6 (4.0%)
Cervix cancer	1 (0.6%)
Gall bladder cancer	2 (1.2%)
Hepatic cancer	1 (0.6%)
Renal cancer	2 (1.2%)
Pancreas cancer	1 (0.6%)
Melanoma	2 (1.2%)
Prostate cancer	1 (0.6%)
Esophageal cancer	1 (0.6%)
Not confirmed	3 (2.0%)
Primary cancer status	
Progression disease	102 (69.4%)
Stable, partial regression	40 (27.2%)
Complete regression	5 (3.4%)
Extracranial metastasis	
Yes	91 (61.9%)
No	56 (38.1%)
Characteristics of brain metastasis	
Presentation type	
Metachronous	82 (55.8%)
Synchronous	65 (44.2%)
Number of metastasis	
Median (range)	2.0 (1–12)
Single	61 (41.5%)
2 - 5	58 (39.5%)
>6	28 (19.0%)
Largest lesion volume (cc)	
Median (range)	5.1 (0.05-59.9)
<5	71 (48.3%)
5-13	55 (37.4%)
13<	21 (14.3%)
Infratentorial/brain stem	
Involvement	
No	95 (64.6%)
Yes	52 (35.4%)
Following WBRT	
Yes	13 (8.9%)
No	134 (91.1%)
Sequential systemic chemotherapy	
Yes	55 (37.4%)
No	92 (62.6%)
Systemic score	
SIRS score	
Median	4.0
0 - 3	42 (28.6%)
4 - 7	105 (71.4%)
GPA score	
Median	1.5
0 - 1.5	77 (52.4%)
2 - 4	70 (47.6%)
RPA class	
II	126 (85.7%)
III	21 (14.3%)
BSBM score	
0 - 1	58 (39.5%)
2 - 3	89 (60.5%)

The enrolled patients were grouped using the four scoring or classification systems evaluated in this study (RPA, SIR, GPA, BSBM). Within the RPA classification, 126 patients (85.7%) were assigned to class II, and the remaining 21 patients were assigned to class III. In the SIR system, 93 patients (63.3%) had a score of 3–5, 22 patients (15.0%) had a score of 1 or 2, and 32 patients (21.8%) had a score > 6. These patients were subsequently classified into low (score 1–3, n = 42) and high (score ≥ 4, n = 105) SIR groups. In the GPA scoring system, 18 patients (12.2%) had a score < 1, 59 (40.1%) had a score between 1 and 2, and the remaining 70 (47.6%) had a score ≥ 2. These patients were thus classified into low (score < 2, n = 77) and high (score ≥ 2, n = 70) GPA groups. In the BSBM scoring system, the majority of the patients had a score of 2 (49.7%) or 1 (35.6%); the remaining patients had a score of 0 (4.1%) or 3 (10.9%). These patients were classified into low (score 0–1, n = 58) and high (score 2–3, n = 89) BSBM groups.

### Overall survival and prognostic factors

**Figure 1:**
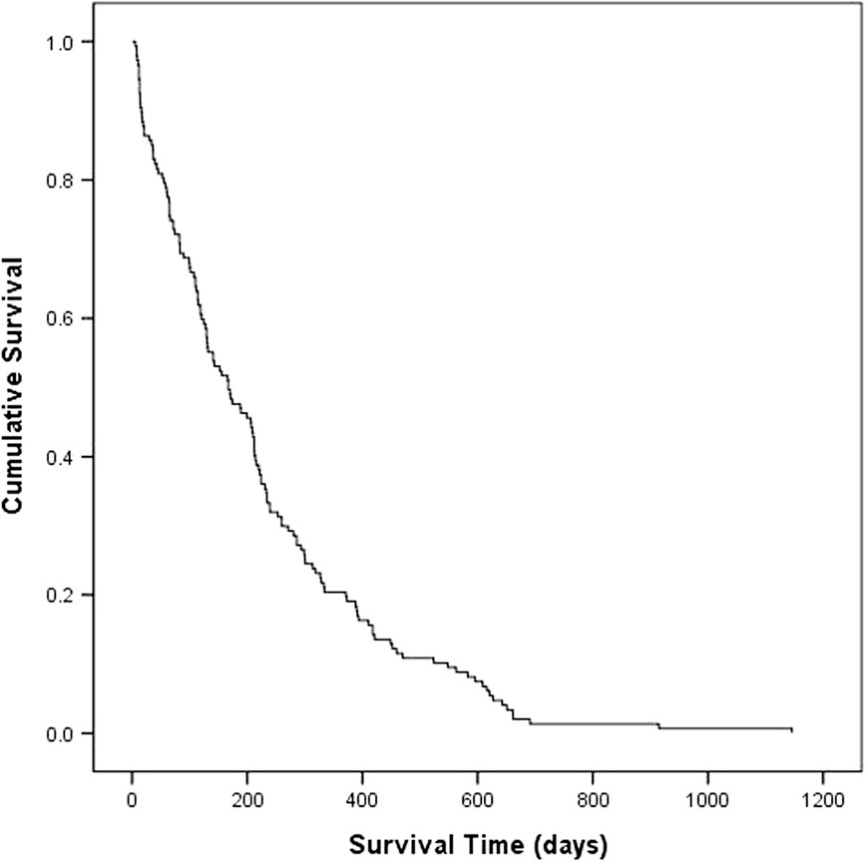
**Overall survival (OS) of 147 elderly patients with brain metastases.** Median OS was 167 days (95% CI: 108.4–225.6). The survival time of 53.1% of the patients was 0.5 years; in 20.4% it was 1 year, and in 1% it was 2 years.

The median OS of the 147 patients who eventually died after GKRS was 167 days (95% confidence interval [CI]: 108.4–225.6 days, Figure 1). The OS rates at 6 months and 1 year were 60.4% and 29.4%, respectively. The cause of death in 120 patients was progressive systemic cancer or related complications (e.g., acute respiratory failure, hepatic failure); 18 patients died as a consequence of brain metastases; and 5 patients died from factors not associated with systemic cancer or brain metastasis, including suicide and myocardial or cerebral infarction. In the remaining four patients the cause of death was not specified.

**Figure 2:**
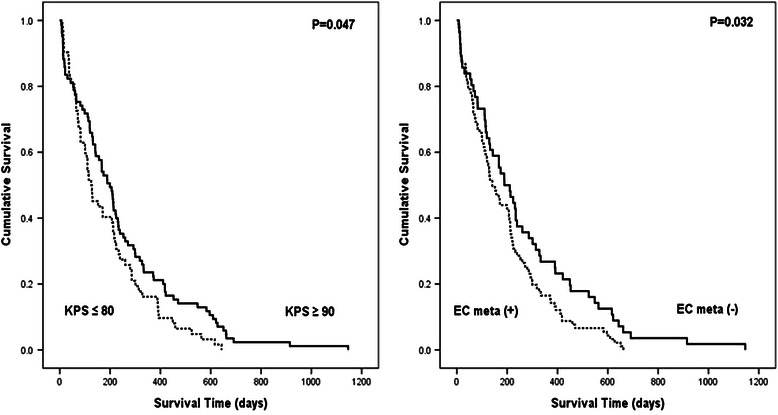
**Kaplan-Meier analyses of overall survival for the 147 study patients according to different predictors (overall comparison was estimated using a log-rank test).***Left:* Survival curve for KPS, *Right:* Survival curve for extracranial metastasis.

**Figure 3:**
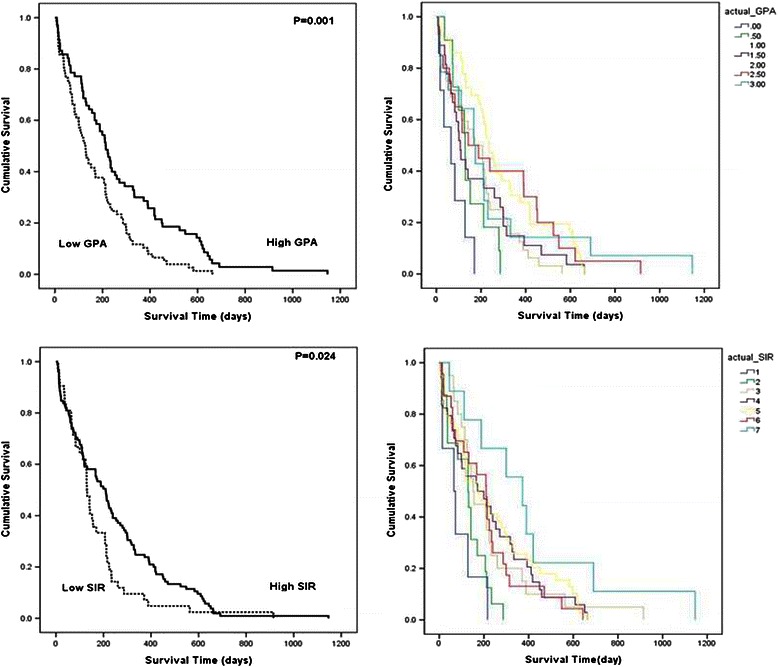
**Kaplan-Meier analyses of overall survival for the 147 study patients according to the different scoring systems (overall comparison was estimated using a log-rank test).***Upper:* Survival curve for GPS, *Lower:* Survival curve for SIR.

The results of statistical analyses of several characteristics of systemic cancer and brain metastasis possibly associated with survival time are summarized in Table 3. KPS (Figure 2, *left*), primary cancer type, and extracranial metastasis (Figure 2, *right*) showed statistical significance in univariate analysis. Of these, KPS and extracranial metastasis were also statistically significant in the multivariate analysis. In addition, a definitive relationship between survival duration after GKRS and the SIR and GPA scores at the time of diagnosis for brain metastasis was determined (Table 3 and Figure 3). Patients with a high SIR score (≥4) had a significantly longer survival time than patients with a low SIR score, as shown in univariate analysis (209 ± 24.7 days *vs.* 130 ± 7.0 days, *P* = 0.024; Figure 3, *lower*). In fact, the duration of survival increased with an increasing SIR score (median survival time of 65, 129, 152, 171, 174, 210, and 373 days for scores of 1–7, respectively, *P* = 0.004). Within the GPA scoring system, patients with a high GPA score (≥2) survived longer than those with a low GPA score (213 ± 22.0 days *vs.* 128 ± 14.9 days, *P* = 0.001; Figure 3, *upper*), and GPA score correlated positively with survival duration (median survival time of 65, 129, 171, 107, 234, 143, 167 for a score of 0, 0.5, 1.0, 1.5, 2.0, 2.5, and 3.0, respectively, *P* = 0.002).

**Table 3:** **Univariateand multivariate analyses for survival predictors in the elderly with brain metastasis after GKRS**

Variable	No. of patients	Survival days (median±SD)	Univariate analysis	Multivariate analysis		
			*P*value	HR	95% CI	*P*value
Characteristics of systemic cancer						
Sex			0.115	ND		0.259
Female	39	212 ± 86.8				
Male	108	156 ± 26.4				
Age			0.780	ND		0.554
70 - 75	80	199 ± 26.3				
≥75	67	130 ± 23.5				
KPS			0.047	1.539	1.089-2.173	0.014
≤80	62	125 ± 19.7				
≥90	85	199 ± 21.2				
Origin cancer			0.032	ND		0.070
Lung	117	171 ± 27.9				
Non-lung or ND	30	110 ± 20.5				
Primary cancer status			0.488	ND		0.980
Non-PD	45	171 ± 27.6				
PD	102	141 ± 32.1				
Extracranial metastasis			0.032	0.633	0.445-0.900	0.011
No	56	189 ± 35.5				
Yes	91	141 ± 19.9				
Characteristics of brain metastasis						
Presentation type			0.289	ND		0.646
Synchronous	65	141 ± 21.1				
Metachronous	82	188 ± 26.3				
Number of metastasis			0.247	ND		0.235
Single	61	209 ± 25.7				
Multiple	86	141 ± 17.7				
Volume of the largest lesion (cc)			0.179	ND		0.410
<13	126	167 ± 30.1				
≥13	21	171 ± 59.5				
Infratentorial/brainstem involvement			0.411	ND		0.363
No	95	156 ± 26.1				
Yes	52	188 ± 42.7				
Sequential systemic chemotherapy			0.257	ND		0.148
No	92	143 ± 18.7				
Yes	55	212 ± 26.5				
Scoring system						
RPA class			0.613		NA	
II	126	167 ± 30.5				
III	21	170 ± 58.7				
SIR score			0.024		NA	
Low (1–3)	42	130 ± 7.0				
High (4–7)	105	209 ± 24.7				
GPA score			0.001		NA	
Low (0–1.5)	77	128 ± 14.9				
High (2–4)	70	213 ± 22.0				
BSBM score			0.273		NA	
Low (0–1)	58	141 ± 17.1				
High (2–3)	89	207 ± 21.7

Multivariate analysis of the important characteristics of systemic cancer with respect to the four scoring systems assessing survival duration identified low GPA score as the most powerful independent factor of short survival (hazard ratio 1.756, 95% CI 1.252–2.456, *P* = 0.001, Table 4).

**Table 4:** **Multivariate analysis for prognostic factors**

Prognostic factors	Multivariate analysis		
	HR	95% CI	*P*value
Male	ND		0.679
≥75 yrs	ND		0.200
KPS≤80	ND		0.223
Non-lung origin	ND		0.073
Presence of extracranial metastasis	ND		0.646
Low SIR score (<4)	ND		0.282
Low GPA score (<2)	1.756	1.252-2.456	0.001

### Prognostic factors favoring longer survival after sequential systemic chemotherapy

Considering the morbidity and side effects associated with chemotherapy in elderly patients, and especially those with terminal cancer, in this study it was important to identify the prognostic factors favoring longer survival after sequential systemic chemotherapy following GKRS. However, among the patients analyzed in this work, sequential chemotherapy for systemic cancer after GKRS did not confer a survival benefit (212 ± 26.5 days *vs*. 143 ± 18.7 days in non-treated patients, *P* = 0.257) regardless of the prognostic variable or scoring system used in the analysis (data not shown).

## Discussion

A cross-national comparison performed in 2000 showed that the proportion of individuals age ≥ 65 years was 12.6 to 18.1% [4], with the proportion predicted to reach 20–28% by 2030 [16]. Along with the growing size of the older population, the incidence of brain metastasis in elderly patients diagnosed with cancer has been rising for several reasons, including the longer survival of patients with a previously diagnosed localized cancer, and the improved detection of metastatic tumors by more sensitive imaging techniques. Nonetheless, in the majority of patients with malignant primary or metastatic brain tumors, age is an important prognostic factor [15,17].

For several decades, WBRT was the treatment of choice for metastatic brain tumors [18]. However, its use in elderly patients was hindered by impaired postoperative functional or cognitive status [9] and poor social services support [19,20] of treated patients. Moreover, although the prophylactic role of WBRT in some cancers has been demonstrated [21], prolonged treatment duration with multiple fractions may not be possible in elderly patients. In a comparison of SRS and WBRT, patients receiving SRS had better OS rates [22]. The preferred use of SRS is that it achieves repeated control of the target lesion without risk of detrimental neurocognitive effects after the therapy [8,23]. Because of its few side effects, GKRS is an excellent treatment option for patients with metastatic brain tumors, including elderly patients. Other advantages of GKRS are that it is minimally invasive, substantially reduces hospitalization time, is relatively inexpensive, and is associated with minimal pain and post-treatment complications [24].

Well-known prognostic scoring systems used to assess patients treated with GKRS for brain metastases are RPA, SIR, BSBM, and GPA, which were created from databases containing 65–1200 patients with brain metastases from a variety of primary tumors [12-15]. GPA was developed to address the limitations of the three other scoring systems; specifically, RPA and BSBM do not consider the number of metastases; RPA, BSBM, and SIR require estimation of the degree of control of the systemic disease, including a primary malignancy, which leads to inconsistencies due to variation in the type and timing of imaging tests. The SIR takes into account treatment factors, such as the volume of the largest lesion at the time of radiosurgery, to predict outcome before treatment decisions are made [25-27]. In the GPA, components of the other scoring systems that are difficult to quantify, such as the control of extracranial disease, were removed as part of the general removal of treatment-related factors such that treatment choice rather than treatment result was reflected [26,27]. The GPA system considers different combinations of diagnosis-specific prognostic factors, and thus better predicts the outcome that can be expected in elderly patients treated with various therapeutic options [28]. The KPS is included in several meaningful scoring systems; in other studies targeting elderly patients, it was shown to be the strongest predictor of prognosis [11].

To identify prognostic factors specific for elderly patients, it is necessary to examine their clinically based prognostic scores and then compare those values with other prognostic scores. Minniti et al. [7] reported that patients with a KPS > 70 and stable extracranial disease had significantly longer survival. Kim et al. [29] found that survival was significantly influenced by the number of brain metastases at the time of SRS, and the primary lung tumor type of the patients. However, while these studies identified KPS, extracranial disease, and the number of brain metastases as significant factors for survival, they did not specify which scoring system most accurately predicted survival in elderly patients with brain metastasis treated with GKRS. A recent report suggested the use of the modified RPA to select favorable candidates for GKRS, even among patients age 80 years and older [30]. In our study, KPS ≥ 90 and no extracranial metastasis at the time of brain-metastasis diagnosis were the most important factors predicting survival. Although in the multivariate analysis both the GPA score and the SIR were statistically significant in predicting survival, the GPA score may be the more powerful independent prognostic factor because it takes into account both the KPS and the presence of extracranial metastasis. Many oncologists and neurosurgeons may hesitate to recommend aggressive treatment for brain metastasis in elderly cancer patients. The results of this and previous studies support the use of GKRS in patients age 70 years and older and even in those 80 years and older. However, although chemotherapy for primary cancer increased the survival of elderly patients according to some studies [31], in our series there were no significant differences between GKRS and GKRS followed by chemotherapy.

## Conclusions

This study was based on a retrospective investigation; thus, selection bias due to missed cases was a possibility. Additionally, there was no information on the quality of life or the control of treated lesions in patients who underwent GKRS. Nevertheless, our results identified extracranial metastases and KPS as independent prognostic factors for survival in elderly patients with brain metastasis treated with GKRS. Among the scoring systems analyzed in this study, the GPA was the most powerful and most specific prognostic scoring system. These are important considerations that influence treatment choice and patient outcome and should be taken into account in therapeutic decision-making.

## Abbreviations

BSBM: Basic score for brain metastases
GKRS: Gamma knife radiosurgery
GPA: Graded prognostic assessment
KPS: Karnofsky performance status
RPA: Recursive portioning analysis
SIR: Score index for radiosurgery
